# Institutional Notes and News

**Published:** 1911-04-22

**Authors:** 


### Institutional Notes and News.

**A Strange Position.**

A most critical state of affairs was reported to a meeting of the Calverley (Yorks) Hospital Board at a meeting held on Wednesday last week. Alderman Wade, the Chairman, remarked that if a fire broke out at the hospital they would be absolutely helpless, as they could not get the necessary force of water from the Bradford Corporation mains. No one will doubt the serious import of such a declaration who remembers that the disastrous fire at Leeds Infirmary last October was due to a similar cause. In The Hospital of October 15 (page 82) we remarked : "It is no use organising your staff unless you have a ready supply of water. . . People, not only at Leeds, are apt to assume a supply as in the nature of things." We had not the officials at Calverley in our minds just then, but we might well have done so, for a proposal that a deputation should interview Mr. Watson, the Waterworks Engineer to the Bradford Corporation, was eventually "left over for the present." The adoption of such a course as that would appear to lay the members of the board open to the severest criticism. The unexpected often happens, as it did at Leeds on October 7, 1910, and the chief consideration of the governing body of a hospital should be to see that due precautions are taken, and that nothing, and certainly not water, is left to chance.

**The Present Situation at Leeds Infirmary.**

Mr. Charles Lupton's speech, as chairman of the annual meeting, gives a capital summary of the present situation at Leeds. To the comments on it which were made in this column last week we now add these extracts that are more particularly interesting in view of the purchases of land that have just been completed. In the last year's report a reference was made to the changes which were needed in the Infirmary building, and during the last year we have been considering these changes a great deal. On the top of our considerations came the fire which occurred in October. We had been led to think that the Fire Brigade being next door we were safe from danger of any kind. But it is perfectly evident that that is not the case, and that we ought to take further precautions to render the building as safe as such a building can be made. As a result of these consultations we decided to go to the public and ask them for a sum of money which would enable us to carry out the plans we have in view, which may be formulated as follows : That we should modernise the existing wards in the institution; that we should build four new operating theatres; that we should provide additional accommodation for our nurses; that we should build two new wards; that we should make additions to the pathological department; additions to the out-patients' department; provide new boilers and other articles of that kind; a proper safeguard against fire; and last, but not of least importance, that we should collect sufficient money to give us a surplus out of which we could draw to meet additions to our annual expenditure, which would be the result of the larger accommodation which we should have to work. In a hospital which has been built nearly fifty years it is necessary that there should be changes which have to be made to bring the hospital up to full modern conditions. If you make a close inspection of the hospital you will notice that it was built much more for able-bodied patients than is the plan of to-day. For instance, the doorways of many of the original bath-rooms were so constructed that it is impossible to carry a patient through them unless you carry him in a perpendicular position. In many of the wards that has now been altered, but not very effectively, and now we are proposing to bring the wards absolutely up to date. So urgent have these alterations been that already we have altered three of the wards without waiting for this fund, which we hope will supply us with the money to do the rest. Luckily for us the ideas of the original builders of the Infirmary were extremely good, and the general plan of the hospital has been excellent. I do not think if we were to build a new hospital to-day it is likely that there would be any considerable change in the ground plan, and that is a great asset. In 1870, which was the first full year of work, there were altogether 470 operations on in-patients. In 1909 we had 4,075 operations upon in-patients, and in the same year we had 3,527 operations upon out-patients under anæsthetics, which is an entirely new thing as compared with the old times. In the first year we had one theatre, latterly we have had two, but these two are very inadequate for the work they have to do, and operations are going on from early in the morning until late at night almost continuously. In Manchester they have one theatre for every 50; in Newcastle, one for every 59; in Leeds we have one for every 135. Additional nurses' accommodation is really only adding more rooms to provide places for the nurses who will be required by the additions to the building itself. —This speech should be read in the light of the Report published in The Hospital of January 29, 1910.

**Progress at the Kensington Hospital.**

A serious attempt is being made to advance the efficiency and interests of the Kensington and Fulham General Hospital, and the success attained up to the present is seen in the satisfactory results of the appeal which was issued last August by Lord Claud Hamilton, the then newly appointed president. It is known that the institution has passed through a trying time of recent years, and one of its obvious difficulties has been its appearance from the street. The present front is not felt to be suitable from the false idea of the size of the building which it gives. A Coronation appeal therefore is being issued by which it is hoped to raise £5,000 to rebuild the front of the hospital. The hospital is a Jubilee institution, having been founded in 1887, and therefore Coronation year means more to it than to others. A Ladies' Guild has also been started, under distinguished presidency, and so Lord Claud Hamilton's term of office is being characterised with energy.

**A Successful Memorial at Norwich.**

Norwich must certainly congratulate itself upon the success of its memorial to King Edward. The wards dedicated to his name, which were opened in February at the Norfolk and Norwich Hospital, are now steadily at work, and what is equally important are secure in the strength of the endowment of £5,000, which was contributed by the executive committee of the Norfolk and Norwich Memorial Fund. This sum of money, it will be remembered, was handed over by Dr. E. E. Blyth, the chairman of the memorial executive committee, on the day of the opening of the wards. Improvement, however, is not likely to stop here, and the building of a new theatre will probably be undertaken which will complete the record of recent progress which the appointment of a bacteriologist, and the addition of balconies has certainly marked. A bust of King Edward by Mr. Albert Bruce-Joy has been placed in the entrance hall.

